# Predictive and prognostic value of circulating nucleosomes and serum biomarkers in patients with metastasized colorectal cancer undergoing Selective Internal Radiation Therapy

**DOI:** 10.1186/1471-2407-12-5

**Published:** 2012-01-04

**Authors:** Yvonne Nadine Fahmueller, Dorothea Nagel, Ralf-Thorsten Hoffmann, Klaus Tatsch, Tobias Jakobs, Petra Stieber, Stefan Holdenrieder

**Affiliations:** 1 Institute of Clinical Chemistry, University Hospital, Munich - Grosshadern, Germany; 2 Institute of Clinical Radiology, University Hospital, Munich - Grosshadern, Germany; 3 Clinics of Nuclear Medicine, University-Hospital, Munich-Grosshadern, Germany; 4 Institute of Radiological Diagnostics, Hospital of the Technical University, Dresden, Germany; 5 Clinics of Nuclear Medicine, Municipial Hospital Karlsruhe, Karlsruhe, Germany; 6 Department of Diagnostic and Interventional Radiology, Hospital Brothers of Charity, Munich, Germany; 7 Institute of Clinical Chemistry and Clinical Pharmacology, University Hospital, Bonn, Germany

**Keywords:** Colorectal cancer, Selective Internal Radiation Therapy, Therapy response, Nucleosomes, Cancer biomarker

## Abstract

**Background:**

Selective Internal Radiation Therapy (SIRT) is a new and effective locoregional anticancer therapy for colorectal cancer patients with liver metastases. Markers for prediction of therapy response and prognosis are needed for the individual management of those patients undergoing SIRT.

**Methods:**

Blood samples were prospectively and consecutively taken from 49 colorectal cancer patients with extensive hepatic metastases before, three, six, 24 and 48 h after SIRT to analyze the concentrations of nucleosomes and further laboratory parameters, and to compare them with the response to therapy regularly determined 3 months after therapy and with overall survival.

**Results:**

Circulating nucleosomes, cytokeratin-19 fragments (CYFRA 21-1), carcinoembryonic antigen (CEA), C-reactive protein (CRP) and various liver markers increased already 24 h after SIRT. Pretherapeutical levels of CYFRA 21-1, CEA, cancer antigen 19-9 (CA 19-9), asparate-aminotransferase (AST) and lactate dehydrogenase (LDH) as well as 24 h values of nucleosomes were significantly higher in patients suffering from disease progression (N = 35) than in non-progressive patients (N = 14). Concerning overall survival, CEA, CA 19-9, CYFRA 21-1, CRP, LDH, AST, choline esterase (CHE), gamma-glutamyl-transferase, alkaline phosphatase, and amylase (all 0 h, 24 h) and nucleosomes (24 h) were found to be prognostic relevant markers in univariate analyses. In multivariate Cox-Regression analysis, the best prognostic model was obtained for the combination of CRP and AST. When 24 h values were additionally included, nucleosomes (24 h) further improved the existing model.

**Conclusion:**

Panels of biochemical markers are helpful to stratify pretherapeutically colorectal cancer patients for SIR-therapy and to early estimate the response to SIR-therapy.

## Background

Colorectal cancer is the third most common cancer in women and men worldwide [1] and accounts for almost 9% of all cancer deaths in the USA [2]. At time of detection of the primary tumor, 15-20% of the patients will already present with liver metastases, another 20% will develop these metastases following treatment of the primary tumor [3]. In 20% of these patients the liver will be the only site of metastases at the time of death [4]. Despite new treatment options nowadays, patients with distant metastases of colorectal cancer just have a five-year survival rate of 11% [2]. Resection of colorectal liver metastases is a potential cure, but unfortunately only a minority of patients (10-15%) is considered as candidates for resection [5]. New local therapy options such as radiofrequency ablation (RFA) and cryotherapy are often performed when surgical resection is not feasible anymore. But also in these therapies, local recurrence rates are directly related to the diameter of the lesions treated and considerably increase in lesions over 4 cm [6,7]. In disseminated liver metastases, surgical resection as well as RFA and cryotherapy cannot be applied anymore. In chemotherapy-refractory patients, Selective Internal Radiation Therapy (SIRT) is a valuable treatment option. Healthy liver tissue is very sensitive to radiation limiting the dosage of external beam radiation to 30-35 Gy to avoid possibly lethal radiation induced liver disease [8]. As liver metastases tend to obtain their blood supply rather from the hepatic artery than from the portal vein [9], microspheres loaded with Yttrium 90 administered through the hepatic artery directly damage the tumor and relatively spare the radiation sensible liver tissue [10]. Thereby an average tumor dosage of 200-300 Gy is applied [11]. A study in metastasized colorectal cancer patients comparing SIRT and systemic chemotherapy consisting of fluorouracil and folinic acid with chemotherapy alone showed a significant improvement in progression-free and overall survival associated with SIRT, both for the total population studied as well as for those patients with disease limited to the liver [12]. However, some earlier studies reported that there was an increase in toxicity with the use of SIRT [13]. Undesirable side effects of SIRT are rare and due to misguided radiating microspheres into the stomach causing gastrointestinal ulcers [14] and into the lungs leading to radiation pneumonitis [15]. To prevent these undesirable side effects, an angiography is performed prior to SIRT to evaluate the individual vascular anatomy and to determine the appropriate placement for the catheter tip. At the end of this procedure, 80-100 Mbq Technetium 99-macroaggregated albumin (Tc-99 m MAA) are administered [16]. As these gamma radiation emitters have the same particle size compared to the resin microspheres, they allow to predict microsphere distribution and quantify hepato-pulmonary-shunting by doing a scintigraphy shortly after the injection [17].

Up to now, there are only rare reports of small studies on factors predicting the therapy outcome in colorectal cancer patients with liver metastases undergoing SIR treatment. Only recently, Dunfee BL et al. reported that response on imaging one month after radioembolization based on World Health Organisation criteria may be a favourable prognostic marker [18]. Having a new powerful but also potentially toxic treatment for liver metastases at hand, prognostic markers are urgently needed to choose pretherapeutically the best treatment available for every patient. Furtheron, early information on the efficacy of the therapy during the first days after SIRT application would be highly appreciated as therapy may be intensified e.g. by chemotherapy or new biological drugs - all the more as the regular staging of the patients by imaging is only done about 3 months after SIRT application.

For both purposes, circulating biochemical markers in the blood are supposed to be most appropriate as that are related to both cancer biology and therapy efficacy. Furtheron, they can be measured non-invasively and cost-efficiently also enabling serial determinations.

Nucleosomes are cell death markers and have been shown to be useful in the early estimation of chemotherapy response in lung cancer patients [19-22], as well as in patients suffering from other solid cancers [23-26]. In the present study we determined nucleosomes and other cell death parameter like cytokeratin-19 fragments (CYFRA 21-1), lactate dehydrogenase (LDH), the colorectal tumor related markers carcinoembryonic antigen (CEA) and cancer antigen 19-9 (CA 19-9) [27], the C-reactive protein (CRP) and classical liver parameters in the serum of patients undergoing SIRT in order to identify markers for predicting therapy response and indicating prognosis.

## Materials and Methods

### Patients

In the present study cohort, 49 colorectal cancer patients suffering from liver metastases (33 males and 16 females, median age 62.6 years, range 35-77 years) treated with SIR therapy at the University Hospital Munich-Grosshadern between May 2006 and May 2008 were prospectively and consecutively included in the study. All patients had primarily been treated with surgical resection and adjuvant chemotherapy (fluoruracil, folinic acid, oxaliplatin and/or irinotecan) and developed metachronous liver metastases lateron. Median time from initial diagnosis to SIRT was 25.1 (5.7-140) months. Patient characteristics are summarized in (Table 1).

**Table 1:** Characteristics of patients with colorectal cancer and liver metastases undergoing SIR therapy

	Median	Range
**Age**	62.6 years	35.3 - 77.9 years

**Time to SIRT after primary diagnosis**	25 months	6-140

	**Number**	**Percentage (%)**

**Patients**	49	100

**Gender**		
Female	16	32.7
Male	33	67.3

**Localisation of primary tumor**		
Colon	11	22.5
Sigma	22	44.0
Rectum	16	32.5

**Chemotherapies before SIRT**		
FOLFOX	42	85.7
FOLFIRI	47	95.9

**Therapy response**		
REM	9	18.4
SD	5	10.2
PD	35	71.4
Progression defining		
liver metastases	19	54.3
extrahepatic manifestation	6	17.1
deceased	10	28.6

One year survival*		
Yes	22	45.8
No	26	54.2

	**Median**	**95% Conf. Interval**

**Overall survival**	8.8 months	5.1-18.1

* one patient lost to follow-up

Inclusion criteria were good performance status (ECOG ≤1) and no other therapy options available for the treatment of the liver metastases. Prior to radioembolization, all the patients were assessed with standard blood tests, whole-body combined positron emission computed tomography (PET-CT), magnetic resonance imaging (MRI) of the abdomen and diagnostic hepatic angiography with application of Technetium 99-macroaggregated albumin (Tc-99 m MAA) followed by thoracic and abdominal perfusion gamma scintigraphy to predict microsphere distribution [17].

Exclusion criteria were previous external beam radiation of the liver and recent treatment with capacitebine [28], significant tumor activity outside the liver, evidence of insufficient liver function (bilirubin > 2 mg/dL, alanine aminotransferase (ALT) and aspartate aminotransferase (AST) > 150 U/L, albumin < 3 mg/dl), platelet count < 50,000/μL, portal vein occlusion and significant hepato-pulmonary shunting > 20% detected in the Tc-99 m MAA scan.

The study was approved by the local ethics committee and written informed consent for additional blood collection and data acquisition was obtained from each patient before radioembolization.

### Treatment procedures

All SIRT procedures were performed under angiographic control (Multistar TOP and Axiom Artis dTA, Siemens, Munich, Germany) and under local anaesthesia. Using Seldinger technique, a 4-French catheter was introduced via the femoral artery. After prophylactic coiling of the gastroduodenal artery and, if necessary, of the right gastric artery and other small visceral vessels, the catheter tip was placed distal of the cystic artery at an identical position as during Tc-99 m MAA application. The dosage of SIR-spheres^® ^was titrated to the calculated extent (*Activity [GBq] = BSA - 0,2 + tumor volume/total liver volume; BSA [m^2^] = 0,20247 × height [m] ^0,725 ^x weight [kG] ^0,425^*) over a time period of 30 to 45 min and administered separately with approximately two thirds of the dose directed to the right lobe. To avoid retrograde embolization of non-target areas due to back spill, sterile water followed by contrast media was flushes alternatedly to the microspheres [16].

### Classification of response to therapy

In all patients, staging investigations consisting of PET-CT and MRI of the abdomen were performed within a median of 94 (range 71-125) days after SIRT to evaluate the response to therapy. As there is currently no accepted standard for the evaluation of PET-CT images for solid tumors [29], response to therapy was determined, as generally conducted in daily routine, by comparing the follow-up with pretherapeutical images. Definition of "progression" was death before staging, the occurrence of new tumor manifestations in the liver or in other organs according to the RECIST criteria for solid tumors [30] with either augmentation in tumor activity in PET-CT, or increase of the tumor diameter ≥20%. 14 of 49 patients did not meet these criteria and thus were classified as non-progressive (5 stable disease, 9 partial remission). 10 patients deceased before the investigations could be performed.

### Sample collection and assays

Blood samples were collected prospectively before therapy, three, six, 24 and 48 hours (h) after SIRT. Subsequently they were centrifuged at 3,000xG for 15 min within one to two hours after venous puncture. After stabilization by adding 10 mM EDTA, sera were stored at -80°C. Prior to the determination of nucleosomes, samples were thawed, homogenized and diluted 1:4 with an incubation buffer. The courses of nucleosomes of each patient were determined within one run of the enzyme immunoassay to minimize the methodical variance.

Quantification of nucleosome concentrations in serum was done by the Cell Death Detection Elisa^plus ^of Roche Diagnostics (Mannheim, Germany). Two monoclonal mouse antibodies, which are directed against histones and DNA, respectively, catch the nucleosomes specifically. The anti-histone antibodies fix the complexes to the microtiter plate, while the anti-DNA antibody, which is labelled with peroxidase, reacts with the 2,2'-azino-di-(3-ethylbenzthiazolin-sulfonate) substrate. The resulting color development is proportional to the amount of nucleosomes which are captured in the antibody sandwich and enables the photometric quantification of nucleosomes in ng/mL according an established standard [31].

In addition, we determined CYFRA 21-1, CA 19-9 and CEA in all blood samples by Elecsys 2010, Roche Diagnostics. LDH, CRP, AST, ALT, bilirubin, gamma-glutamyl-transferase (GGT), alkaline phosphatase (AP), amylase, lipase and choline esterase (CHE) were measured by high-end analyzer AU 2700 (Olympus Diagnostics, Hamburg, Germany) in all but the three and six hours post treatment samples.

### Statistics

Concentrations of all measured markers before, three, six, 24 and 48 h after SIRT as well as their differences compared to pretherapeutic levels were considered for statistical evaluation.

Concerning their response to therapy, patients with partial remission and stable disease were combined into the 'no progression' group. They were compared to patients who suffered from progressive disease.

For the assessment of significance between marker levels in therapy response groups, the Wilcoxon test was used. For analysis of survival time, the marker values were separated into equal quartiles which were used as cut-offs in Kaplan-Meier analysis and log-rank test.

In multivariate analyses, all parameters before and 24 h after SIRT significant in Kaplan-Meier analyses were included into Cox-Regression models. Generally all values were logarithmized and entered the multivariate evaluation as continuous variables. As for some parameters few values were missing (pretherapeutic values: LDH (N = 6), CHE (N = 3), CYFRA 21-1 (N = 1), AP (N = 3), CA 19-9 and CEA (N = 2), nucleosomes (N = 1); 24 h values: LDH (N = 2), CA 19-9, CEA and nucleosomes (N = 1), they were replaced by the medians of the cohort. Calculations were performed without and with missing values and no difference in the results were seen. In a first step, only pretherapeutic levels of all univariatly significant parameters (nucleosomes, CYFRA 21-1, AST, GGT, AP, CHE, LDH, CRP, CA 19-9 and CEA) were combined and all possible combinations of two or three parameters were tested on their prognostic value for overall survival. Second, 24 h values were additionally taken into account in the same setting. Only those models with all variables being significant were considered. Subsequently, performance of the models was compared by Akaike Information Criterion (AIC) to identify the most valuable models.

A *P*-value of < 0.05 was considered statistically significant. All calculations were performed by software of SAS (version 9.2, SAS Institute Inc., Cary, N.C., USA).

## Results

Already 24 h after application of SIRT, concentrations of circulating nucleosomes, CYFRA 21-1, CEA, liver enzymes and bilirubin were increased whereas GGT, AP, CHE and activity of lipase and amylase decreased significantly. LDH, CRP and CA 19-9 remained stable during the first 24 h and significantly increased 48 h after SIRT. While this effect was most obvious in cell death biomarkers such as nucleosomes and CYFRA 21-1 it was less visible in surface cancer biomarkers such as CEA and CA 19-9 (Table 2).

**Table 2:** Levels of biomarkers before, 3, 6, 24 and 48 h after application of SIRT

	Reference value (95th perc. of healthy)	0 hours	3 hours	6 hours	24 hours	48 hours
		Median	Median	Median	Median	Median
		Range	Range	Range	Range	Range
**Nucleo-somes **[ng/mL]		**190.5**	**197.6**	**237.4**	**758.5**	**596.0**
	**< 36**	5.0-1624	34.8-2501	40.7-2064	21.7-4334	51.7-2547

**CYFRA 21-1** [ng/mL]		**10.9**	**9.3**	**8.8**	**28.6**	**27.5**
	**< 2.2**	0.8-246.0	0.9-240.0	1.1-230.0	1.0-811.0	1.5-638.0

**CEA** [ng/mL]		**330.0**	**343.5**	**356.5**	**351.0**	**433.0**
	**< 3.4**	2.0-31620	1.9-33010	1.9-16980	1.6-36120	1.6-37550

**CA 19-9 **[U/mL]		**40.0**	**41.6**	**49.7**	**48.7**	**56.4**
	**< 37**	2.4-11530	2.3-24400	2.2-8748	2.4-18550	2.3-17000

**LDH** [U/L]		**407.0**			**486.5**	**585.0**
	**< 250**	178.0-3068			143.0-2923	142.0-3585

**CRP** [mg/dL]		**1.3**			**1.3**	**4.6**
	**< 0.5**	0.1-20.3			1.3-21.8	0.1-16.3

**CHE** [kU/L]		**7.3**			**6.3**	**5.9**
	**5.0-13.3**	2.8-11.1			2.8-11.1	5.9-10.5

**AST** [U/L]		**52.0**			**74.5**	**68.5**
	**< 33**	24.0-190.0			15.0-339.0	19.0-317.0

**ALT** [U/L]		**34.5**			**46.0**	**48.5**
	**< 35**	15.0-268.0			14.0-178.0	13.0-136.0

**Bilirubin** [mg/dL]		**0.7**			**1.0**	**1.2**
	**< 1.0**	0.3-1.9			0.4-2.2	0.4-2.8

**AP** [U/L]		**160.0**			**148.0**	**156.0**
	**< 135**	81.0-742.0			63.0-655.0	61.0-630.0

**GGT** [U/L]		**183.5**			**165.5**	**168.5**
	**< 38**	33.0-712.0			31.0-577.0	31.0-653.0

**Amylase** [U/L]		**58.0**			**44.0**	**46.5**
	**< 100**	20.0-136.0			20.0-429.0	20.0-519.0

**Lipase** [U/L]		**26.0**			**18.0**	**20.0**
	**< 60**	3.0-263.0			3.0-585.0	3.0-495.0

**Figure 1:**
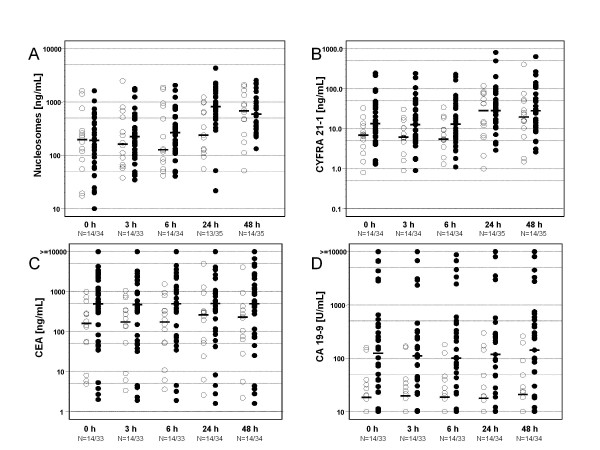
**Distribution of serum concentrations of nucleosomes (**A**), CYFRA 21-1 (**B**), CEA (**C**) and CA 19-9 (**D**) comparing therapy response groups**. ○ Responder; ● Non-Responder.

When evaluating therapy response 3 months after SIRT, 14 patients out of 49 showed stable disease or partial remission (no progression) while the remaining 35 patients either deceased within time to staging (N = 10), developed progressive disease in the liver (N = 19) or in other organs (N = 6). (Table 1) Concerning therapy response, the pretherapeutical levels of CA 19-9, CEA, CYFRA 21-1, LDH, CHE and AST showed significant differences between both groups. Although pretherapeutical values of circulating nucleosomes differed not between responder groups, higher nucleosomes values were measured 24 h after SIRT in patients with progressive disease as compared with non-progressive patients (*p *= 0.0034). CHE and CA 19-9 were the only markers which showed significant differences concerning therapy response for every time point examined (Figure 1, Table 3).

**Table 3:** Correlation of biomarkers with therapy response

		Responder		Non-Responder		
Marker	Time [hours]	Median	Range	Median	Range	p-Value
**Nucleosomes** [ng/mL]	0	**198.5**	17.4-1624	**174.7**	5.0-1617	0.991
	24	**239.1**	55.6-1225	**844.8**	21.7-4334	**0.003**
	48	**677.2**	51.7-2095	**594.9**	133.3-2547	0.851

**CYFRA 21-1** [ng/mL]	0	**6.9**	0.8-32.8	**14.0**	1.3-246.0	**0.031**
	24	**28.4**	1.0-118.0	**30.8**	2.9-811.0	0.493
	48	**19.6**	1.5-404.0	**28.5**	2.6-638.0	0.293

**CEA** [ng/mL]	0	**162.0**	4.0-986.0	**557.0**	2.0-31620	**0.037**
	24	**265.5**	2.6-5011	**540.5**	1.6-36120	0.087
	48	**229.5**	2.2-4073	**524.0**	1.6-37550	0.063

**CA 19-9** [U/L]	0	**18.6**	2.4-159.0	**148.0**	2.4-11530	**0.001**
	24	**17.9**	2.5-298.0	**158.0**	2.4-18550	**0.004**
	48	**20.9**	2.4-260.0	**187.5**	2.3-17000	**0.002**

**LDH** [U/L]	0	**349.0**	178.0-544.0	**529.5**	220.0-3068	**0.011**
	24	**450.0**	143.0-1117	**532.0**	148.0-2923	0.522
	48	**572.0**	142.0-1488	**607.5**	168.0-3585	0.434

**CRP** [mg/dL]	0	**0.9**	0.3-5.1	**2.4**	0.1-20.3	0.227
	24	**1.1**	0.1-2.8	**2.8**	0.2-21.8	0.159
	48	**4.4**	0.1-16.3	**4.6**	0.3-15.1	0.507

**AST** [U/L]	0	**37.5**	24.0-94.0	**56.0**	24.0-190.0	**0.037**
	24	**111.0**	32.0-169.0	**69.0**	15.0-339.0	0.782
	48	**85.0**	33. 0-179.0	**66.0**	19.0-317.0	0.912

**ALT** [U/L]	0	**38.0**	15.0-63.0	**30.0**	15.0-268.0	0.658
	24	**52.0**	26.0-131.0	**37.0**	14.0-178.0	0.108
	48	**54.0**	23.0-112.0	**34.0**	13.0-136.0	0.113

**GGT** [U/L]	0	**183.5**	44. 0-432.0	**189.0**	33.0-712.0	0.782
	24	**168.5**	46.0-391.0	**164.0**	31.0-577.0	0.965
	48	**177.5**	60.0-406.0	**170.0**	31.0-653.0	0.903

**AP** [U/L]	0	**153.0**	89.0-569.0	**193.0**	81.0-742.0	0.559
	24	**156.0**	78.0-588.0	**151.0**	63.0-655.0	0.707
	48	**156.0**	77.0-504.0	**164.0**	61. 0-630.0	0.674

**Bilirubin** [mg/dL]	0	**0.7**	0.3-1.4	**0.8**	0.3-1.9	0.438
	24	**0.9**	0.4-1.7	**1.0**	0.4-2.2	0.920
	48	**1.1**	0.5-2.4	**1.2**	0.4-2.8	0.799

**CHE** [kU/L]	0	**8.3**	4.9-11.1	**6.5**	2.8-10.8	**0.017**
	24	**7.1**	5.1-11.1	**5.7**	2.8-10.4	**0.009**
	48	**6.9**	4.5-10.5	**5.3**	2.3-9.7	**0.006**

**Amylase** [U/L]	0	**64.5**	20.0-105.0	**54.0**	24.0-136.0	0.346
	24	**45.5**	21.0-79.0	**44.0**	20.0-429.0	0.833
	48	**50.5**	20.0-58.0	**46.0**	23.0-519.0	0.842

**Lipase** [U/L]	0	**27.0**	3.0-149.0	**25.0**	3.0-263.0	0.725
	24	**17.5**	4.0-33.0	**18.0**	3.0-585.0	0.773
	48	**16.5**	3.0-42.0	**22.0**	3.0-495.0	0.330

*P*-values (by Wilcoxon-test) are shown for the various response groups.

**Figure 2:**
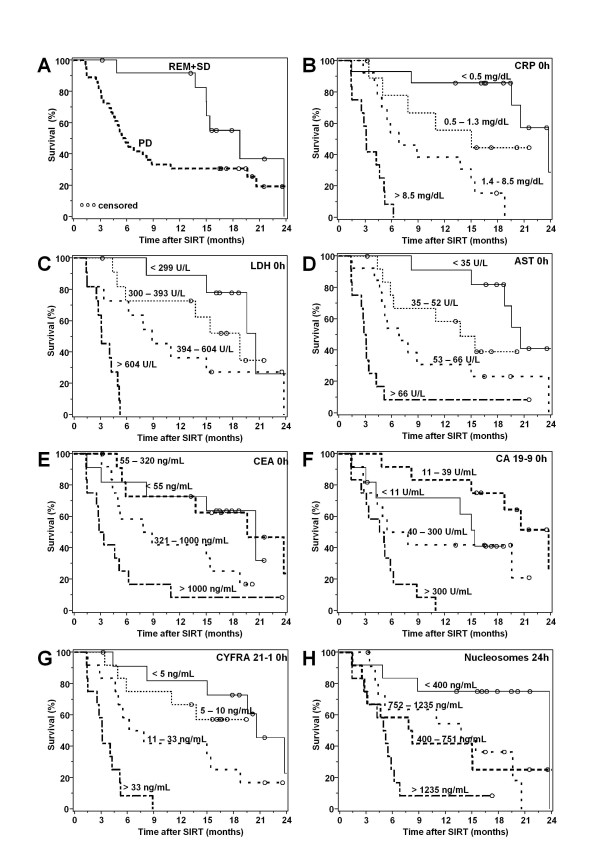
**Kaplan Meier curves showing the overall survival of patients according to response to therapy (**A**) (REM = remission; SD = stable disease; PD = progressive disease) and quartiles of CRP (**B**), LDH (**C**), AST (**D**), CEA (**E**), CA 19-9 (**F**), CYFRA 21,1 (**G**) before therapy as well as of nucleosomes (**H**) 24 h after SIRT**.

There was a significant correlation between therapy response and survival time in this setting (*s *= 0.034). (Figure 2A).

In additiona, several laboratory markers were found to be associated with the overall survival: When medians, 25th and 75th percentiles were used as cutoffs for the various markers, values before and 24 h after SIRT of CEA, CA 19-9, CYFRA 21-1, AST, GGT, AP, CHE, LDH, Amylase and CRP as well as nucleosomes (24 h) showed to be significant prognostic markers in univariate analyses (Figure 2B-H, Table 4).

**Table 4:** Kaplan-Meier analyses of all parameters with median survival and 95% confidence interval before and 24 h after SIRT

Biomarker	Time	Quartile 1		Quartile 2		Quartile 3		Quartile 4		p-value
		
		**Median survival **[months]	95% Confi. Interval	**Median survival **[months]	95% Confi. Interval	**Median survival **[months]	95% Confi. Interval	**Median survival **[months]	95% Confi. Interval	Log-rank
**Nucleosomes **[ng/mL]	0 h	< 83		83-190		191-415		> 415		0.1275
		**17.3**	1.5 -	**8.2**	4.6 -	**13.8**	3.2-23.8	**6.0**	1.5-15.4	
	
	24 h	< 384		384-751		752-1232		> 1232		**0.0004**
		**23.8**	4.9-23.8	**8.0**	2.5 -	**13.8**	4.0-20.6	**5.1**	1.4-6.1	

**CYFRA 21-1** [ng/mL]	0 h	< 5.3		5.3-10.0		10.0-32		> 32.8		**< .0001**
		**20.6**	8.2 -		4.9 -	**7.0**	2.8-18.8	**3.1**	1.4-5.1	
	
	24 h	< 13		13-28		29-74		> 74		**0.0009**
		**20.6**	8.2-23.8	**6.3**	3.4 -	**6.2**	3.1 -	**4.6**	1.4-15.0	

**CEA** [U/L]	0 h	< 55		55-320		321-1041		> 1041		**0.0002**
		**20.6**	3.1 -	**19.6**	5.6 -	**8.3**	4.2-18.8	**3.1**	1.4-6.1	
	
	24 h	< 85		85-351		352-982		> 982		**0.0002**
		**20.6**	8.2-23.8	**19.6**	4.2 -	**5.5**	2.5-8.8	**4.9**	1.5-15.0	

**CA 19-9** [U/L]	0 h	< 10		10-40		41-285		> 285		**0.0006**
		**15.0**	3.1 -	**23.8**	8.2 -	**6.6**	2.8 -	**4.9**	1.4-6.2	
	
	24 h	< 10		10-48		49-285		> 285		**0.0096**
		**15.4**	3.1 -	**17.8**	4.2-23.8	**6.6**	2.8 -	**5.5**	1.4-11.0	

**CRP** [mg/dL]	0 h	< 0.5		0.5-1.3		1.4-8.3		> 8.3		**< .0001**
		**23.8**	19.6 -	**14.4**	3.4 -	**6.8**	4.4-15.0	**3.1**	1.4-5.1	
	
	24 h	< 0.7		0.7-1.3		1.4-6.4		> 6.4		**< .0001**
		**23.8**	11.0-23.8		4.7 -	**8.8**	5.8-15.4	**3.0**	1.4-5.1	

**LDH** [U/L]	0 h	< 299		299-393		394-604		> 604		**< .0001**
		**20.6**	8.2 -	**15.4**	4.7 -	**8.8**	1.5-23.8	**3.2**	1.5-5.0	
	
	24 h	< 336		336-486		487-878		> 878		**0.0002**
		**23.8**	8.2 -	**11.0**	4.4 -	**6.8**	4.6-16.8	**2.8**	1.4-15.0	

**AST** [U/L]	0 h	< 35		35-52		53-66		> 66		**< .0001**
		**20.6**	15.0 -	**13.8**	5.0 -	**7.3**	4.6-15.0	**3.0**	1.4-4.2	
	
	24 h	< 48		48-74		75-135		> 135		**0.0004**
			11.0 -	**6.3**	4.6-19.6	**5.6**	2.8-15.4	**3.4**	1.5-15.0	

**ALT** [U/L]	0 h	< 26		26-34		35-50		> 50		0.0773
		**18.8**	5.2 -	**7.8**	3.1 -	**13.8**	5.1-15.4	**3.8**	1.5-23.8	
	
	24 h	< 30		30-46		47-64		> 64		0.0824
		**20.6**	5.2 -	**5.5**	4.4-15.0	**15.0**	2.6-19.6	**4.1**	1.5 -	

**CHE** [kU/L]	0 h	< 5.8		5.8-7.3		7.4-8.4		> 8.4		**0.0013**
		**5.1**	1.5-6.2	**5.9**	2.8 -	**8.2**	4.0-19.6	**28.8**	11.0-23.8	
	
	24 h	< 5.1		5.1-6.3		6.4-7.6		> 7.6		**0.0002**
		**4.7**	1.4-6.8	**5.4**	2.5-20.6	**23.8**	4.4 -	**18.8**	8.2 -	

**GGT** [U/L]	0 h	< 110		110-183		184-282		> 282		**0.0034**
		**16.7**	5.9 -	**18.8**	4.4-20.6	**11.0**	2.8-15.4	**4.4**	2.5-7.8	
	
	24 h	< 94		94-165		166-274		> 274		**0.0101**
		**17.3**	6.8 -	**12.5**	4.4-20.6	**15.0**	2.8 -	**4.8**	2.5-7.8	

**AP** [U/L]	0 h	< 114		114 - 160		161 - 321		> 321		**< .0001**
		**20.6**	8.2 -	**18.8**	4.6 - 23.8	**6.2**	3.2 -	**4.2**	1.5 - 5.5	
	
	24 h	< 98		98 - 148		149 - 265		> 265		**< .0001**
		**23.8**	8.2 -	**13.8**	4.6 - 19.6	**7.8**	3.4 - 18.8	**3.7**	4.1 - 6.2	

**Bilirubin **[mg/dL]	0 h	< 0.5		0.5 - 0.7		0.8 - 1.2		> 1.2		0.2393
		**14.4**	4.4 -	**11.0**	2.8 -	**18.8**	5.1 -	**4.4**	1.5 - 15.0	
	
	24 h	< 0.8		0.8 - 1.0		1.1 - 1.8		> 1.8		0.3335
		**10.3**	4.0 - 19.6	**15.4**	4.6 -	**5.9**	2.5 -	**8.2**	1.5 - 23.8	

**Amylase** [U/L]	0 h	< 44		44 - 58		59 - 75		> 75		**0.0102**
		**5.2**	1.4 - 18.8	**7.5**	3.2 - 19.6	**17.4**	2.5 - 23.8		5.8 -	
	
	24 h	< 35		35 - 44		44 - 63		> 63		**0.0030**
		**5.2**	3.2 - 15.4	**6.2**	3.1 -	**15.0**	2.8 -	**20.6**	1.5 -	

**Lipase** [U/L]	0 h	< 13		13 - 26		27 - 38		> 38		0.5314
		**5.7**	1.5 -	**18.8**	2.8 -	**8.8**	4.0 -	**13.0**	3.4 - 19.6	
	
	24 h	< 10		10- 18		18 - 28		> 28		0.6226

In multivariate Cox regression analyses, all combinations of two or three parameters including all pretherapeutic values of univariately relevant prognostic markers were tested resulting in a variety of possible prognostic models. The lowest and thus best AIC (Akaike Information Criterion) value with all parameter still significant was attained for the combination of CRP with AST. This model had even higher prognostic power than any combination of three pretherapeutic parameters. It has to be emphasized that a similar AIC value was obtained by the combination of CRP and LDH as well suggesting that AST is exchangeable with LDH. An even lower AIC and thus a better prognostic information was achieved when, in addition, 24 h values of all relevant markers were considered in Cox analyses. Then, the combination model of pretherapeutic levels of CRP and AST and nucleosomes 24 h after SIRT yielded best prognostic information. (Table 5)

**Table 5:** Multivariate prognostic models for survival time

	Parameter	Coefficient	Hazard-Ratio	95%-Conf Interval	Chi-Square	p-value	AIC
**Model 1 Only pretherapeutic values**	**CRP** **(0 h)**	0.469	**1.6**	1.3-2.0	16.5	**< 0.0001**	187
	
	**AST** **(0 h)**	0.917	**2.5**	1.4-4.4	10.3	**0.0013**	

**Model 2 Pretherapeutic and 24 h values**	**CRP** **(0 h)**	0.323	**1.4**	1.1-1.8	6.6	**0.0104**	176
	
	**AST** **(0 h)**	1.351	**3.9**	2.0-7.5	16	**< 0.0001**	
	
	**Nucleosomes **(24 h)	0.565	**1.8**	1.2-2.5	10.4	**0.0012**	

## Discussion

The first experiences of radioembolization with Yttrium 90 for patients suffering from liver metastases that originated from colorectal cancer were published in 1964 by Arial and coworkers [32]. However, as radiation leakage into non-targeted tissues was a non-controllable problem, the therapy was abandoned until the late 1980s, when new resin and glass microspheres were developed [33] and pretherapeutical Tc-99 m MAA scans were able to identify high risk patients for undesirable side effects [17]. Until recently, SIRT was used as a salvage therapy, when all other treatment options like surgical treatment, chemotherapy and local ablation were exhausted [12,16,33,34]. In recent years, some studies have been published that demonstrated promising results with positive benefit for patients when SIRT was combined with chemotherapy [12,34]. In February 2009, a worldwide multicenter study started comparing combined SIRT with FOLFOX versus FOLFOX alone as a first-line therapy in colorectal cancer patients with liver metastases too extensive for surgery [35].

If those investigations lead to promising results, SIRT may be included into earlier treatment concepts in the therapeutic management of colorectal cancer patients. However, there is still only rare information on potential prognostic parameters for patients undergoing SIR therapy [18]. Such prognostic factors would be highly desirable for the pretherapeutic selection of those patients who will most benefit from SIR therapy. Other subgroups of patients who have no or only limited benefit from radioembolization could be identified [33] to avoid unnecessary side effects and high expenses without gaining a benefit for the patient.

Furtheron the efficacy of SIRT should be early estimated - if possible already during the first days after the application - in order to potentially intensify the therapy strategy i.e. by completing SIRT by chemo- or biological therapies. As serum-based biomarkers are easily accessible, cheap and can be determined in serial measurements, they are ideal tools to pretherapeutically predict the therapy outcome enabling an effective patient stratification for SIR treatment and to monitor local and systemic biochemical SIRT effects when determined repeatedly during the very early phase after SIRT application.

In this study, a broad panel of biomarkers relevant for various features of tumor biology and treatment effects was monitored: CEA and CA 19-9 are well-established oncological biomarkers used in the follow-up care and prognosis of colorectal cancer patients [36,37] and CEA levels are observed to be strongly elevated in cancer patients suffering from liver metastases [38]. Cell death markers nucleosomes, CYFRA 21-1 and LDH reflect pathophysiological processes in strongly proliferating tumors as well as the cellular and immunological effects of SIRT on the tumor and the organism as a whole. The liver markers AST, ALT, GGT, bilirubin, AP and CHE and the pancreatic enzymes amylase and lipase indicate the more organ-specific alterations and potential side effects such as temporary pancreatits due to misguided microspheres [39]. CRP is frequently used as a marker for inflammation, but may also serve as prognostic tool in cancer patients [40].

The concentrations of all examined parameters showed significant alterations at least 24 or 48 h after SIRT. These results were expected for all markers reflecting high cell turnover by a rapid release and a short half-life of several hours such as nucleosomes and CYFRA 21-1 [41]. This phenomenon was less pronounced for the cancer biomarkers CEA and CA 19-9 that are known to be more stable surface parameters with minor increases and longer half-life times in blood of 2 to 5 days. Therefore those markers normally are only considered to be analyzed in two to three months intervals during the follow-up of patients with colorectal cancer [42].

Concerning overall survival of colorectal cancer patients undergoing liver surgery, pretherapeutic CEA has earlier been reported to be a powerful predictor [37,38]. Our results are consistent with those findings as pretherapeutical CEA levels clearly distinguished between patient groups with different therapy responses and were predictive for survival time.

In addition, CA 19-9 values before, 24 and 48 h after SIRT significantly indicated the later therapy outcome and survival in our setting. Similarly, CA 19-9 levels have earlier been shown to predict survival in patients who underwent surgical resection of colorectal cancer [37] although it is officially not recommended for follow-up care of colorectal cancer patients [36].

While many cancers are characterized by high cellular turnover including massive cell proliferation and cell death, the concentration of the cell death end products nucleosomes in serum varies between different types of cancers and also between individuals [43,44]. Interestingly, levels of nucleosomes were found to be higher in advanced tumor stages and in patients with metastasis of bowel cancer that may be attributed to the increasing amount of dysfunctional cells [44]. Thus we expected to find high rates of nucleosomes before, as well as during therapy. The pretherapeutic median value of nucleosomes (191 ng/mL) was considerably higher than in healthy individuals (36 ng/mL) [23]. Already three and six hours after SIRT slowly increasing values of nucleosomes were detected in most patients followed by a rapid and significant rise after 24 h; these elevated levels lasted also until the second day after therapy. Most notably, increased nucleosome levels 24 h after SIRT indicated significantly poor therapy response and reduced survival time. On a first view, these results appear irritating as an acute and extended induction of apoptosis after irradiation was reported to lead to favourable therapy responses particularly in some cancers treated in early stages [45] and thus should result in high nucleosome levels. However, in several settings of cytotoxic radio- and chemotherapies of patients with advanced stage cancers, the contrary association was observed with high nucleosome levels during the first days after therapy and unfavourable outcome such as in studies on patients with advanced colorectal [25,26], pancreatic [24], non-small cell [19,21,22] and small cell lung cancer [20]. In those settings, non-responding advanced tumors may have more aggressive features with high cell turnover and a better blood supply leading to more effective release of nucleosomes into the blood or a less effective elimination of nucleosomes from the circulating due to a weakened or impaired immune system.

Similar kinetics as for nucleosomes were also observed for other cell death markers like CYFRA 21-1 and LDH. Cytokeratin-19 fragments are part of the cell cytoskeleton and are released during pathologies with high cell death rates [41]. CYFRA 21-1 is known as a sensitive tumor marker that is valuable in supporting the differential diagnosis, therapy monitoring and estimation of non-small cell lung cancer and some other cancer entities, particularly in advanced stages [41,46,47]. CYFRA 21-1 was at high levels already before start of therapy and considerably increased 24 and 48 h after SIRT. Interestingly, only the pretherapeutical CYFRA 21-1 values were helpful in indicating poor therapy response while for the estimation of overall survival all examined time points yielded significant results. It correlated clearly with LDH (R > 0.85 for every single point examined) which also was of important predictive and prognostic value.

CRP is known as a sensitive but non-specific inflammatory marker which is induced by IL-6 in the liver. In addition, it provides strong prognostic information for overall survival in patients with colorectal cancer undergoing surgery [40] or adjuvant chemotherapy [48]. Also in our setting, high CRP values had a strongly unfavourable prognostic relevance. Interestingly, high AST levels were as well associated with poor prognosis. That may be the result of pretherapeutically compromised liver tissue or a postembolization syndrome after normal liver tissue damage by SIRT induced ischemia. However, alterations of ALT levels and concentrations of other liver parameters were less pronounced and were not as strongly associated with prognosis of patients.

Joining all univariately significant results for overall survival into multivariate testing of possible double and triple marker combinations revealed CRP and AST as best model of independent prognostic markers available before start of the therapy. When 24 h values were added as well, nucleosomes 24 h further improved the prognostic power of the existing model. This illustrates that pretherapeutic CRP and AST as well as 24 h nucleosome levels cover different pathological processes and therapy induced effects and are additive to each other in their prognostic information.

Although the number of patients included in this study seems to be quite limited, it has to be emphasized that all patients had similar pathology, received a standardized therapy in a speciality center for SIR treatment, were monitored with a very strictly defined protocol during the early post-treatment period and were correlated with regular, high standard imaging methods after 3 months and follow- up of overall survival. Considering these preconditions and the low rate of missing values, this study constitutes a quite informative approach on the relevance of biomarker levels or changes in the prediction and prognosis of colorectal cancer patients undergoing SIRT. Three and six hours values were included in the program not to miss any relevant time point mirroring the immediate effects of the therapy. However, while most biomarkers remained stable or even showed temporary decreases at these times points, they increased considerably after 24 and 48 h values and then showed more informative prognostic values in this setting. Similar results have earlier been obtained in the monitoring of colorectal and pancreatic cancer patients during radiochemotherapy [24,25]. It was speculated that the temporary decline could be explained by post-damage cell cycle arrest which was followed by delayed cell death if the therapy induced lesions could not be repaired any more.

As a further strength of the present study, besides the completeness of the data, it has to be pointed out that all preanalytic steps between blood drawing, laboratory processing, stabilisation and deep-frozen storage of the samples and later analysis were under full control of the study personal and followed a strict preanalytic protocol to avoid any processing delay or in-vitro manipulation of the samples. Further, the biomarker analyses were performed by experienced laboratory staff with all samples of individual patients in one test run to minimize potential interassay variations and final interassay cross-plate validations were added as further quality check. Laboratory testings were conducted independently of any clinical data collection. Statistical evaluation was done independently from both laboratory testing and clinical data collection by the statistic section of the study center.

According to current standards, the therapy response was evaluated with PET-CT and MRI in comparison with pretherapeutic images. The staging was performed consistently after two to three months after SIRT - with the exception of a few patients who were evaluated with some delay due to clinical reasons. This staging period is generally chosen to closely monitor the macroscopic changes which often only develop slowly: For example in 35 patients with hepatocellular carcinoma treated with SIRT, WHO responses were only seen in 3 patients after 1 month and increased to 13 patients after half a year [49]. Besides the delay until changes are detectable, CT or MRI staging results often underly technical limitations as alterations of tumor size does not necessarily reflect tumor response to treatment but may be attributed to non-specific effects like haemorrhage and peritumoral oedema that may be misinterpreted as progressive disease [33].

Further evaluation according RECIST criteria only takes into account the change of the largest lesion diameter but not any volumetric measures [30]; further it mirrors only the changes of tumor size but not the more important changes of tumor activity that may have decreased considerably despite stable tumor volume.

An earlier and more precisely evaluation of tumor response is obtained by using PET-CT as it is a functional body-imaging and thus visualizes not just macroscopic but also metabolic changes [50]. Therefore, the results of PET-CT exams were priorized when evaluating the therapy response in this study if there were inconsistencies with MRI results in the staging investigations.

Concerning the evaluation of response to this locoregional liver-confined SIR therapy, it could be debated whether only the changes of the therapy-related organ should be concerned or whether the overall response to therapy including the development of further intra- or extrahepatic metastases and tumor-related death should be respected. As the systemic outcome would be more relevant for the patient and the marker response in the blood would more reflect those systemic changes including the possible growth of pre-existing micrometastases, we chose this endpoint as most informative therapy outcome.

As further endpoint, overall survival was considered. Because the patient sample was quite homogeneous and the clinical characteristics were similar based on the inclusion criteria to this study, only the dosage of the microspheres administered and biomarker levels were examined in relation with survival time. To avoid an overfitting of the prognostic results to the present dataset, e.g. by cutoff optimization, the biomarker levels entered the multivariate analyses as logarithms in a continuous form. As considerable correlations between different biomarkers were observed, for example between AST and LDH, several prognostic scores with similar strength would be possible in Cox regression analyses. To avoid the risk to find one specific model by chance, all possible combinations of two and three markers with pretherapeutic values alone (model 1) and with pretherapeutic as well as 24 h values considered (model 2) were compared by the Akaike Information Criterion (AIC) indicating the strength of a prognostic model. In this way, a combination of two pretherapeutic markers (CRP and AST) was identified as most powerful prognostic model that would be appropriate for the patient stratification for SIR therapy, with a further improvement of the individual prognostic information by adding the determination of nucleosomes 24 h after therapy.

## Conclusion

To our knowledge this prospective single-center study on an homogenous colorectal cancer patient cohort with hepatic metastases treated by SIRT is the first exploratory, and hypotheses-generating approach to identify biomarkers during the early treatment phase for the estimation of prognosis and the early estimation of therapy response that will be validated by further, larger prospective treatment studies.

## Abbreviations

CRC: Colorectal cancer; SIRT: Selective Internal Radiation Therapy; PET-CT: Positron emission tomography - computed tomography; MRI: Magnet resonance imaging; CA 19-9: Cancer antigen 19-9; CYFRA 21-1: Cytokeratin-19 fragments; LDH: Lactate dehydrogenase; CRP: C-reactive protein; CHE: Choline esterase; AST: Aspartate aminotransferase; ALT: Alanine aminotransferase; GGT: Gamma-glutamyl-transferase; AP: Alkaline phosphatase; Gy: Gray; MAA: macroaggregated albumin; Gbq: Giga Becquerel; BSA: Body surface area; AIC: Akaike Information Criterion.

## Competing interests

The authors declare that they have no competing interests.

## Contributions of the authors

YNF, DN, PS, and SH designed the present study and coordinated the logistic process. YNF, RTH, KT and TJ were responsible for recruitment of patients, defined blood sampling and clinical data collection. YNF, PS and SH were responsible for immunoassay measurements. Statistical analysis was performed by DN. YNF, PS, DN and SH were involved in the interpretation of the data and the conception of the manuscript. All authors read and approved the final manuscript.

## Pre-publication history

The pre-publication history for this paper can be accessed here:

http://www.biomedcentral.com/1471-2407/12/5/prepub
